# Optimisation of Sub-30 nm Solid Lipid Nanoparticles Loaded With Docetaxel Produced by the Cold-Burst Method: A Particle Size Optimisation Study

**DOI:** 10.7759/cureus.90291

**Published:** 2025-08-17

**Authors:** Oluwaseun Akinniranye, Anastasia Goryanin, Olusegun Akinniranye

**Affiliations:** 1 Hospital Medicine, Princess Alexandra Hospital, Harlow, GBR; 2 Anesthesiology, Princess Alexandra Hospital, Harlow, GBR

**Keywords:** nano-drug delivery system, nanomedicine, solid lipid nanoparticles, targeted therapy, uro-oncology

## Abstract

Introduction: The cold-burst method presents a novel, energy-efficient, and cost-effective approach for solid lipid nanoparticle (SLN) production compared to traditional methods. It involves simple heating and cooling cycles that can create SLNs below 30 nm in size. Given the limitations of conventional docetaxel (DTX) delivery in cancer therapy, SLNs offer a promising solution for improved bioavailability and reduced toxicity. The achievement of sub-30 nm SLNs is particularly significant, as this size range is known to enhance passive tumour targeting via the enhanced permeability and retention (EPR) effect, promote deeper distribution into solid tumours, and improve cellular uptake. This study aimed to optimise the particle size of DTX-loaded SLNs produced via the cold-burst method.

Method: Formulations utilised Compritol 888 ATO (888) and Precirol ATO 5 (ATO5) as lipid components, stabilised by water-soluble (Tween20, BrijS20) and oil-soluble (monoolein) surfactants. A total of 25 SLN formulations were created by systematically varying parameters including type of lipid, type of water-soluble surfactant, DTX concentration (0-5 wt%, equating to 0-5 mg), total surfactant concentration (2-4 wt%), water-soluble surfactant ratio, and number of heating and cooling cycles. Particle size (mean D50) was determined by dynamic light scattering (DLS) using a Malvern Zetasizer Nano ZS series (Malvern Panalytical, Shanghai, China). Statistical comparisons of mean D50 values were performed using one-way analysis of variance (ANOVA) followed by Tukey's honestly significant difference (HSD) post hoc analysis.

Results: This study's results consistently demonstrated the successful formation of <30 nm DTX-loaded SLNs for both lipids. Drug loading of up to 5 wt% DTX showed no significant difference in particle size when compared to no drug loading. A significant decrease in particle size was observed with increasing total surfactant concentration (2-4 wt%). Both water-soluble surfactants used in this study, Tween20 and BrijS20, facilitated cold bursting and the creation of sub-30 nm SLNs, with BrijS20 yielding significantly smaller nanoparticles across both lipid types. The most pronounced size reduction for 888 SLNs occurred within the first heating and cooling cycle.

Conclusion: These findings highlight the potential of cold-burst-derived sub-30 nm SLNs as an optimised platform. Our work demonstrates the successful optimisation of particle size for DTX-loaded SLNs, laying a foundation for future comprehensive studies towards enhanced DTX delivery and more effective cancer therapeutics. However, the absence of drug loading quantification, in vitro drug release data, and cellular performance assessments limits the conclusions regarding the therapeutic efficacy of these SLNs.

## Introduction

The importance of solid lipid nanoparticles (SLNs)

The concept of SLNs was first presented in the 1990s to blend the characteristics of lipid-based emulsions and nanoparticles [1]. The combination of these two formulations gave SLNs significant potential for clinical use. The tailored system that allows for drug loading and potential targeted delivery through systemic circulation directly stems from the characteristics of nanoparticles. Conversely, their non-toxicity, biocompatibility, and biodegradability are all traits gained from lipid-based emulsions. The combination of all these characteristics makes SLNs a much more attractive drug delivery system than other colloidal drug carriers, like liposomes. Research over the past few decades has shown that SLNs hold many advantages over more traditional colloidal drug carriers, including cheaper mass production, potential for modified drug release and targeted delivery, enhanced drug solubility, increased stability, and the incorporation of both hydrophilic and hydrophobic drugs [2]. The properties and advantages that SLNs possess mean they have great potential in solving numerous clinical challenges. Over the past decade, SLNs have been researched for use in many different clinical therapeutics, with cancer therapeutics being the most researched clinical use for SLNs [3]. With continued research, in the future, SLNs could move towards widespread, cost-effective clinical use. Ultimately, SLNs could help to reduce dosing frequency, improve drug bioavailability, and decrease non-adherence to drug therapy.

Clinical Need for Prostate Cancer Therapeutics

In industrialised countries, prostate cancer is the most common male cancer and the fifth leading cause of cancer-related deaths in men [4]. In the UK, one in eight men will be diagnosed with prostate cancer in their lifetime, and on average, one man dies from prostate cancer every 45 seconds [5]. The number of people dying from prostate cancer increases annually, continually adding more clinical and financial pressure to the NHS. Despite improvements in cancer therapeutics, more men are dying of prostate cancer than ever, highlighting the need for continued research.

SLNs as carriers for docetaxel (DTX)

DTX is a second-generation taxane that has been approved for the treatment of a broad range of cancers, including prostate cancer. Its mechanism of action is inhibiting the assembly of microtubules into mitotic spindles, thereby arresting cancer cell cycling during G2/M. In addition, DTX downregulates the BCL2 gene, often expressed in cancer cells, leading to tumour cell apoptosis. DTX is typically administered intravenously; however, this is normally accompanied by multiple side effects. The known adverse effects of DTX include infusion reactions, myelosuppression, anaemia, and hypersensitivity reactions. Oral administration of DTX would be preferable to intravenous, as oral administration is less costly, non-invasive, and available to outpatients which would improve patient's quality of life [6]. Oral administration of DTX is limited due to first-pass metabolism and DTX's extreme hydrophobicity, both of which result in poor bioavailability [7]. The progression of nanoparticle research makes SLNs a potential oral delivery system for DTX that can improve bioavailability and reduce toxicity. SLNs' lipid core allows hydrophobic drugs, like DTX, to be encapsulated within them, therefore increasing solubility. SLNs have already been shown to increase the lymphatic uptake, intestinal absorption, and oral bioavailability of DTX in rats compared with Taxotere (an intravenous DTX formulation) [8]. Moreover, nanoparticles have been shown to accumulate around tumours through the enhanced permeability and retention (EPR) effect [9]. The EPR effect improves the delivery of nanosized drugs to tumours without the need for specific targeting, further underlining how SLNs can improve the delivery of DTX as a cancer therapeutic.

The cold-burst method

The cold-burst method for SLN formulation is an extremely novel technique, with the first paper detailing it being published in 2020 [10]. Traditionally, nanoparticle emulsions were produced by highly energy-inefficient methods like high-pressure homogenisation and microfluidisers [11]. The cold-burst method was discovered as a new process which is both cost-effective and energy-efficient. Cold burst produces nanoparticles by subjecting lipid particles to controlled heating and cooling cycles. Literature has shown that one heating and cooling cycle resulted in particle diameter decreasing from 100 μm to 0.4 μm [11]. When lipids are surrounded by an aqueous solution, cooling followed by heating results in a phase change that allows the aqueous solution to be dragged into the lipids, causing them to "burst" into smaller nanoparticles [10,11]. The lack of expensive equipment and low energy requirements make the cold-burst method an attractive alternative to previous nanoparticle formation methods.

The potential for a cold burst in SLN creation

Since the cold-burst method is such a novel technique, the literature surrounding it is scarce. The lack of literature about cold burst leaves a lot of room for research into its method, uses, and optimisation. Currently, there is little information regarding lipid-based nanoparticles suitable for drug delivery. This study looks to identify and optimise the size of SLNs that are made of biocompatible lipids, which are also loaded with the cancer therapeutic DTX.

Effect of particle size and size distribution on SLN use

It is well documented that particle size and size distribution are crucial factors for both the clinical use and mass production of SLNs. Specifically in terms of cancer therapeutics, nanocarriers above 150 nm are unable to enter or exit fenestrated capillaries in the tumour microenvironment [12]. Research has suggested that nanocarriers below 200 nm can passively target tumour tissues due to the EPR effect [13]. Furthermore, the majority of therapeutic nanoliposomes are designed to have 50-100 nm diameters to avoid mononuclear phagocyte system-mediated clearance and to prolong blood circulation time [12].

More importantly, it is critical for cancer-targeted nanoparticles to be <30 nm, as particles between 10 and 30 nm enter cells more efficiently and distribute more deeply into solid tumours than larger particles [14]. In addition, particles less than 30 nm are shown to access intracellular targets of tumour cells more effectively [15]. This is especially important for DTX, as its mechanism of action is inhibiting intracellular microtubules.

For mass production, the size distribution of SLNs is an important parameter to optimise. Poor size distribution would require post-production filtering of SLN solutions to ensure the correct size is acquired. Filtering may cause wastage of materials, ultimately making production methods less energy-efficient and more costly.

Currently, few studies focus on clinically viable SLN formulations using low-cost methods. Alongside exploring the feasibility of making <30 nm SLNs using cold burst, this study looks to explore factors which optimise particle size and particle size distribution via the cold-burst method. Due to the scope of this study, distribution metrics were only assessed in an exploratory manner via volume-weighted size distribution graphs.

Objectives

Our primary objective was to achieve and optimise mean D50 ≤30 nm for DTX-loaded SLNs produced by the cold-burst method, with particle size measured by dynamic light scattering (DLS) at 25°C (three replicate runs per sample). To achieve this, the study addressed the following aims in the order of analysis: to quantify the effect of total surfactant concentration (2-4 wt%) on mean D50, to quantify the effect of DTX loading (0-5 wt%) on mean D50, to quantify the size-reducing effect of heating and cooling cycles (0-6) on mean D50, to assess the effect of the Tween20-to-monoolein (water-soluble surfactant-to-oil-soluble surfactant) ratio (2:1-6:1) on mean D50 in Compritol 888 ATO (888) formulations, and to compare water-soluble surfactant types (BrijS20 vs. Tween20) on mean D50.

## Materials and methods

Lipids

To prepare the oil-in-water emulsions, two lipids were used: Precirol ATO 5 (ATO5) and 888. ATO5, purchased from Gattefossé, is a lipid that has been proven to create drug-loaded SLNs [16] and has also been proven to produce SLNs using cold burst [10]. ATO5 consists of glyceryl distearate and glyceryl palmitostearate, which are esters of palmitic (C16) and stearic (C18) acids. 888, purchased from Gattefossé, is a hydrophobic mixture of mono-, di-, and triesters of behenic acid (C22). It has also been shown to successfully create DTX-loaded SLNs [17]. For all SLN formulations, 1 wt% (with respect to 10 g of water, equating to 0.1 g) of either ATO5 or 888 was used.

Surfactants

To stabilise the emulsions, water-soluble and oil-soluble surfactants were used.

For each SLN formulation, the total surfactant concentration ranged between 2 wt% and 4 wt% (with respect to 10 g of water, equating to 0.2-0.4 g).

Water-Soluble Surfactants

BrijS20 was one of the water-soluble non-ionic surfactants used to stabilise the oil-in-water emulsions. It is mostly used for pharmaceutical applications to improve the solubility of drugs, but research has also shown it can create stable nanoparticles [18]. BrijS20 was purchased from Sigma-Aldrich.

Tween20 was the other water-soluble surfactant used to stabilise the oil-in-water emulsions. It is widely used for biochemical applications and has also been shown to create stable SLNs [10]. Tween20 was purchased from Sigma-Aldrich.

For each SLN formulation, the ratio of water-soluble surfactant to oil-soluble surfactant ranged from 2:1 to 6:1. This changed the weight of the water-soluble surfactant depending on the total surfactant concentration. The specific weights are detailed later in the Results section. 

Oil-Soluble Surfactants

Monoolein is an oil-soluble surfactant that was used as the sole oil-soluble surfactant to stabilise the oil-in-water emulsions. From the literature, it is known that monoolein leads to an efficient cold-burst process [11].

High-purity monoolein is expensive, so an analogue grade was purchased, 1-oleoyl-rac-glycerol ~40% (GM40) from Sigma-Aldrich, and the surfactant was purified by the following aqueous ethanol extraction method.

A 10 wt% dispersion of GM40 was prepared that contained 65 wt% ethanol and 35 wt% water. The solution was stirred for two hours at 5°C, before being centrifuged for one hour at 5°C and 5000 revolutions per minute (rpm). Next, the upper phase that contains monoglyceride was decanted and transferred to another bottle. The contents were evaporated for two hours at 50°C. Then the solid phase was redissolved in an aqueous ethanol solution (95 wt% ethanol and 5 wt% water) and stirred for two hours at 5°C. Finally, the solution was centrifuged at 15°C for one hour, the upper phase decanted, and the ethanol evaporated fully at 50°C.

For each SLN formulation, the ratio of water-soluble surfactant to oil-soluble surfactant ranged from 2:1 to 6:1. This changed the weight of the oil-soluble surfactant depending on the total surfactant concentration. The specific weights are detailed later in the Results section. 

DTX

DTX is a chemotherapy drug used to treat various types of cancer. Literature shows that DTX can be loaded into SLNs composed of different materials and formulated by various methods [13].

The amount of DTX used in each SLN formulation was either 0 wt%, 2 wt%, or 5 wt% with respect to the lipid. This would equate to 0 mg, 2 mg, or 5 mg, respectively.

DTX was purchased from Apollo Scientific.

Oil-in-water emulsion formation

To prepare the oil-in-water emulsions, the oily and aqueous phases were prepared separately. The oily phase contained the chosen lipid and DTX. These were measured and placed into the same glass vial before being mixed with a magnetic stirrer and heated on a hot plate. Mixing was performed in a 15 mL glass vial with a 5 mm polytetrafluoroethylene (PTFE) stir bar at 750 rpm for 10 minutes. The lipid was heated to a temperature above its melting range (above 77°C for 888 and above 60°C for ATO5). Heating and mixing continued until the lipid was visibly melted and had fully dissolved the DTX.

The aqueous phase contained water, the chosen water-soluble surfactant, and monoolein. Deionised water was consistently used for all aqueous-phase preparations. These components were measured and placed into a separate glass vial. The aqueous phase was mixed with a magnetic stirrer and heated on a hot plate. Mixing in this vial was also performed with a 5 mm PTFE stir bar at 750 rpm for 10 minutes to ensure the surfactants were visibly dissolved, creating a transparent solution.

The oily and aqueous phases were then poured into the same vial before heating and mixing at a temperature sufficiently high to melt and dissolve all components. Mixing at 750 rpm continued for a further 15 minutes until all components of the oil-in-water emulsion were dissolved. The oil-in-water emulsion was then rapidly cooled in an ice bath (5 ± 1°C) for 30 seconds to solidify the lipid droplets.

For every sample, 10 g of deionised water was used as the continuous phase. Other emulsion components were systematically varied to create a total of 25 SLN formulations.

Heating and cooling cycles

A HighTech cryo-compact circulator from Julabo was used as a water bath to heat the samples and induce cold bursting. Samples were heated at 0.5°C/min from a temperature below to a one above the melting range of the chosen lipid (above 77°C for 888 and above 60°C for ATO5). After heating, the samples were taken out of the cryo-compact circulator and rapidly cooled by complete submersion in an ice bath (5 ± 1°C) for 30 seconds to solidify the SLNs. This rapid cooling procedure was designed to emulate a cooling rate of approximately 2.5°C/s based on prior studies [11]. The temperature was followed during the cooling process, which resulted in a cooling rate of between 2 and 4°C/s. Heating and cooling cycles were repeated variably depending on the SLN formulation that was created.

Particle size and size distribution measurements

Information on particle size and particle size distribution after one to multiple heating and cooling cycles was determined by DLS measurements. DLS measurements were made using a Malvern Zetasizer Nano ZS series (Malvern Panalytical, Shanghai, China). Zetasizer Nano software was used to analyse the results.

Malvern Zetasizer Nano ZS series was operated in non-invasive backscatter mode at a scattering angle of 173°C. Measurements were made in DTS1070 folded capillary cells, at a controlled temperature of 25°C with a five-minute equilibration prior to data acquisition. The dispersant (deionised water) was specified in the software with a refractive index (RI) of 1.333 and viscosity of 0.887 centipoise.

SLN production creates formulations with a range of nanoparticle sizes. As a result, D50 was calculated to represent the particle size of each SLN formulation. It is a percentile value indicating that 50% of all particles in the sample are smaller than the given size. D50 for this experiment measured the diameter of the nanoparticles in nanometres. For each sample, three measurements were performed and averaged, with manual inspection to exclude any outlier runs or air-bubble artefacts. The mean D50 values of the three measurements were used for this study's results.

Only instrument replicates were used for repeat measurements, not independent batch formulations.

Statistical analysis

Data was collected from Zetasizer Nano software version 8.02 and analysed using Microsoft Excel (Microsoft Corp., Redmond, WA, USA).

A one-way analysis of variance (ANOVA) was used to compare the mean D50 values of the SLN samples. Tukey's honestly significant difference (HSD) test was used as a post hoc test to compare different SLN formation variables against each other.

## Results

Table 1 and Table 2 present all 25 SLN formulations that were created for this study. The formulations have been ranked in descending order of smallest mean D50 to largest mean D50.

**Table 1:** Mean D50 values for all SLN formulations that were created for this study. D50: percentile value indicating that 50% of all particles in the sample are smaller than the given size; DTX: docetaxel; SLN: solid lipid nanoparticles; 888: Compritol 888 ATO; ATO5: Precirol ATO 5

Sample number	Oil	DTX%	Water-soluble surfactant	Water-soluble surfactant-to-oil-soluble surfactant ratio	Number of cycles	Total surfactant concentration %	Mean D50 (d.nm)
1	ATO5	5	BrijS20	3:1	3	3	15.48 ± 1.02
2	888	2	BrijS20	3:1	3	4	18.17 ± 0
3	888	5	BrijS20	3:1	6	3	19.41 ± 4.47
4	888	2	BrijS20	3:1	5	3	20.08 ± 1.66
5	888	2	BrijS20	3:1	6	3	21.04 ± 0
6	888	0	BrijS20	3:1	6	3	22.04 ± 7.61
7	888	2	BrijS20	3:1	2	3	23.35 ± 0
8	ATO5	0	Tween20	3:1	3	4	23.52 ± 1.92
9	888	2	BrijS20	3:1	4	3	24.36 ± 0
10	888	2	BrijS20	3:1	3	3	25.64 ± 2.22
11	ATO5	2	Tween20	3:1	3	4	26.02 ± 0
12	888	2	BrijS20	3:1	3	3	27.13 ± 4.80
13	ATO5	5	Tween20	3:1	3	4	28.38 ± 0
14	888	2	BrijS20	3:1	1	3	31.18 ± 2.57
15	888	2	BrijS20	3:1	3	2	32.67 ± 0
16	ATO5	5	Tween20	3:1	3	3	39.83 ± 0
17	ATO5	5	Tween20	3:1	3	3	39.83 ± 2.56
18	ATO5	5	Tween20	3:1	3	2	87.38 ± 0
19	888	2	Tween20	3:1	4	4	99.89 ± 78.23
20	888	2	Tween20	5:1	4	4	102.84 ± 132.24
21	888	2	Tween20	6:1	4	4	106.69 ± 102.46
22	888	2	Tween20	4:1	4	4	110.69 ± 95.61
23	888	2	BrijS20	3:1	0	3	191.50 ± 28.03
24	888	2	Tween20	2:1	4	4	298.60 ± 158.48
25	888	2	Tween20	3:1	3	3	380.90 ± 67.38

**Table 2:** Formulation parameters for all SLN formulations that were created for this study. DTX: docetaxel; SLN: solid lipid nanoparticles

Sample number	Lipid weight (g)	DTX weight (g)	Water-soluble surfactant weight (g)	Oil-soluble surfactant weight (g)
1	0.1000	0.0050	0.2250	0.0750
2	0.1000	0.0020	0.3000	0.1000
3	0.1000	0.0050	0.2250	0.0750
4	0.1000	0.0020	0.2250	0.0750
5	0.1000	0.0020	0.2250	0.0750
6	0.1000	0.0000	0.2250	0.0750
7	0.1000	0.0020	0.2250	0.0750
8	0.1000	0.0000	0.3000	0.1000
9	0.1000	0.0020	0.2250	0.0750
10	0.1000	0.0020	0.2250	0.0750
11	0.1000	0.0020	0.3000	0.1000
12	0.1000	0.0020	0.2250	0.0750
13	0.1000	0.0050	0.3000	0.1000
14	0.1000	0.0020	0.2250	0.0750
15	0.1000	0.0020	0.1500	0.0500
16	0.1000	0.0050	0.2250	0.0750
17	0.1000	0.0050	0.2250	0.0750
18	0.1000	0.0050	0.1500	0.0500
19	0.1000	0.0020	0.3000	0.1000
20	0.1000	0.0020	0.3333	0.0667
21	0.1000	0.0020	0.3429	0.0571
22	0.1000	0.0020	0.3200	0.0800
23	0.1000	0.0020	0.2250	0.0750
24	0.1000	0.0020	0.2667	0.1333
25	0.1000	0.0020	0.2250	0.0750

888 cold bursting

The following results are for SLN formulations where 888 was used as the lipid. 

Effect of Changing the Total Surfactant Concentration on Particle Size

**Figure 1:**
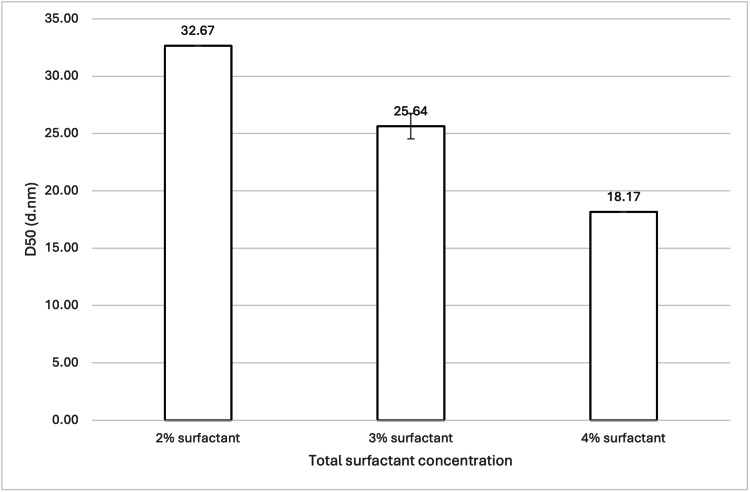
Effect of total surfactant concentration on 888 SLN D50 values. For each group, n = 3. A one-way ANOVA showed a significant difference in the mean D50 values at a 95% confidence interval (p = 0.00002802), with a Tukey's HSD post hoc analysis confirming a significant difference between all three samples (all p values < 0.05). This indicates that increasing total surfactant concentration from 2 wt% to 3 wt% and 4 wt% (with respect to 10 g of water) significantly decreased the 888 SLN particle size. 888: Compritol 888 ATO (solid lipid); SLN: solid lipid nanoparticles; D50: percentile value indicating that 50% of all particles in the sample are smaller than the given size; ANOVA: analysis of variance; HSD: honestly significant difference

**Figure 2:**
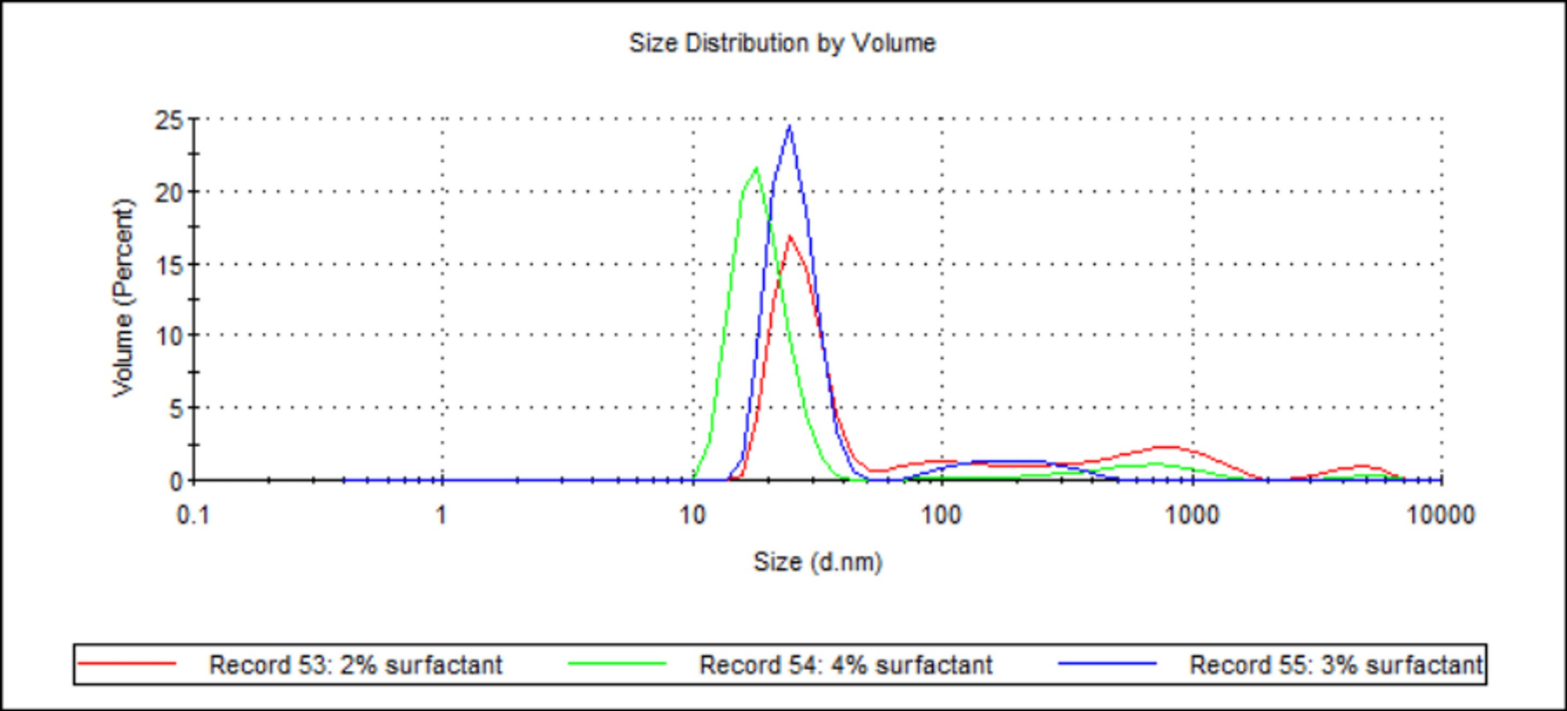
Volume-weighted particle size distributions showing the effect of total surfactant concentration on 888 SLN sizes. For each group, n = 3. This figure displays the volume-weighted particle size distribution profiles for 888 SLN formulations prepared with varying total surfactant concentrations (2 wt%, 3 wt%, and 4 wt%). The x-axis represents the particle size in nanometres (d.nm) on a logarithmic scale, and the y-axis represents the volume percentage of particles at a given size. All three formulations exhibit prominent primary peaks in the nanometre range (between 10 nm and ~40 nm). The 4 wt% and 3 wt% surfactant formulations exhibit very sharp, narrow primary peaks around 15-20 nm, while the 2 wt% surfactant shows a slightly broader peak shifted to a slightly larger size and a more pronounced tail of larger aggregates. 888: Compritol 888 ATO (solid lipid); SLN: solid lipid nanoparticles; d.nm: diameter in nanometres

Three samples (see Table 1 and Table 2; samples 2, 10, and 15) were analysed, with the total surfactant concentration being the sole difference between them. The samples contained either 2 wt%, 3 wt%, or 4 wt% (with respect to 10 g of water) total surfactant concentration. Figure 1 shows the mean D50 values for each sample, while Figure 2 shows the volume-weighted particle size distributions for each sample (derived from Zetasizer Nano software version 8.02).

Effect of Changing the DTX Concentration on Particle Size

**Figure 3:**
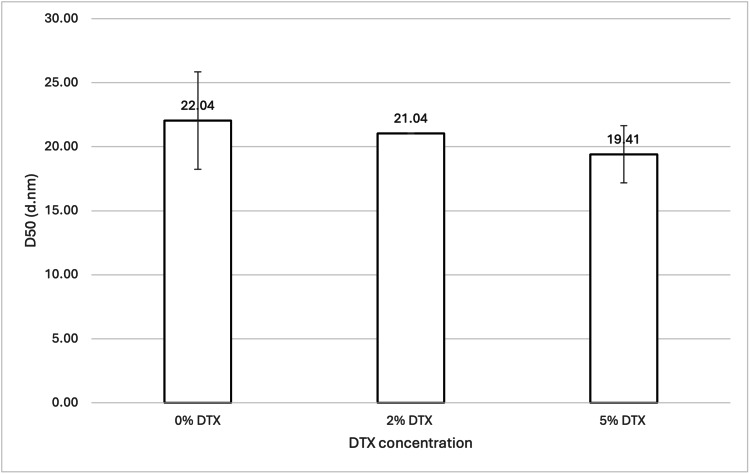
Effect of DTX concentration on 888 SLN D50 values. For each group, n = 3. A one-way ANOVA showed that there was no significant difference in the D50 values, indicating that varying DTX concentrations (0 wt%, 2 wt%, and 5 wt% with respect to 888) did not significantly affect the particle size of 888 SLN samples. DTX: docetaxel; 888: Compritol 888 ATO (solid lipid); SLN: solid lipid nanoparticles; ANOVA: analysis of variance; D50: percentile value indicating that 50% of all particles in the sample are smaller than the given size

**Figure 4:**
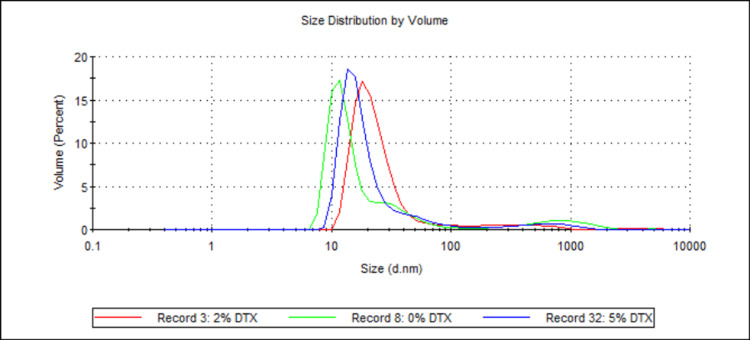
Volume-weighted particle size distributions illustrating the effect of DTX concentration on 888 SLN sizes. For each group, n = 3. This figure presents the volume-weighted particle size distribution profiles for 888 SLN formulations with varying DTX concentrations (0 wt%, 2 wt%, and 5 wt% with respect to 888). The x-axis indicates the particle size in nanometres (d.nm) on a logarithmic scale, and the y-axis represents the volume percentage of particles at a given size. All three formulations show distinct primary peaks in the nanometre range (between 10 nm and ~30 nm), demonstrating the successful formation of sub-30 nm nanoparticles created with up to 5 wt% DTX. DTX: docetaxel; 888: Compritol 888 ATO (solid lipid); SLN: solid lipid nanoparticles; d.nm: diameter in nanometres

Three samples (see Table 1 and Table 2; samples 3, 5, and 6) were analysed, with the total DTX being the sole difference between them. The samples contained either 0 wt%, 2 wt%, or 5 wt% (with respect to 888) DTX. Figure 3 shows the mean D50 values for each sample, while Figure 4 shows the volume-weighted particle size distributions for each sample (derived from Zetasizer Nano software version 8.02).

Effect of the Number of Cycles on Particle Size

**Figure 5:**
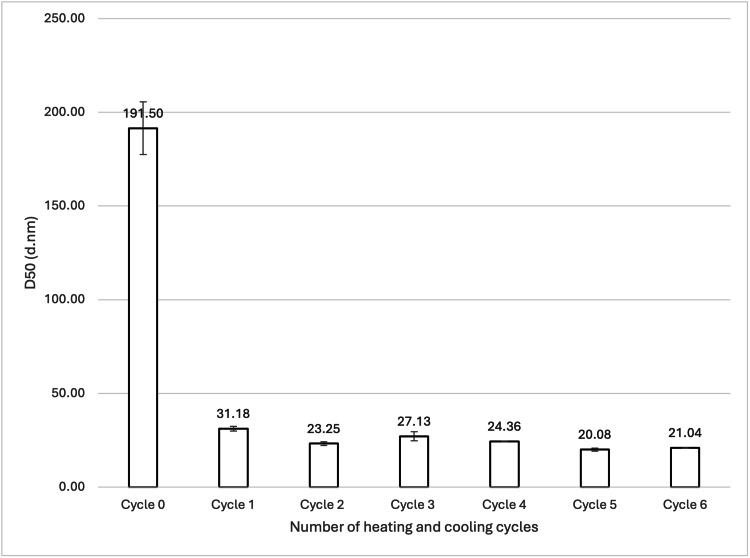
Effect of heating and cooling cycles on SLN D50 values. For each group, n = 3. A one-way ANOVA showed a significant difference in the mean D50 values at a 95% confidence interval (p= 0.00000000009560). A Tukey's HSD post hoc analysis confirmed a significant reduction in SLN particle size at a 95% confidence interval after cycle 0 compared to all subsequent cycles, with no further significant differences observed between cycle 1 and cycle 6. 888: Compritol 888 ATO (solid lipid); SLN: solid lipid nanoparticles; D50: percentile value indicating that 50% of all particles in the sample are smaller than the given size; ANOVA: analysis of variance; HSD: honestly significant difference

**Figure 6:**
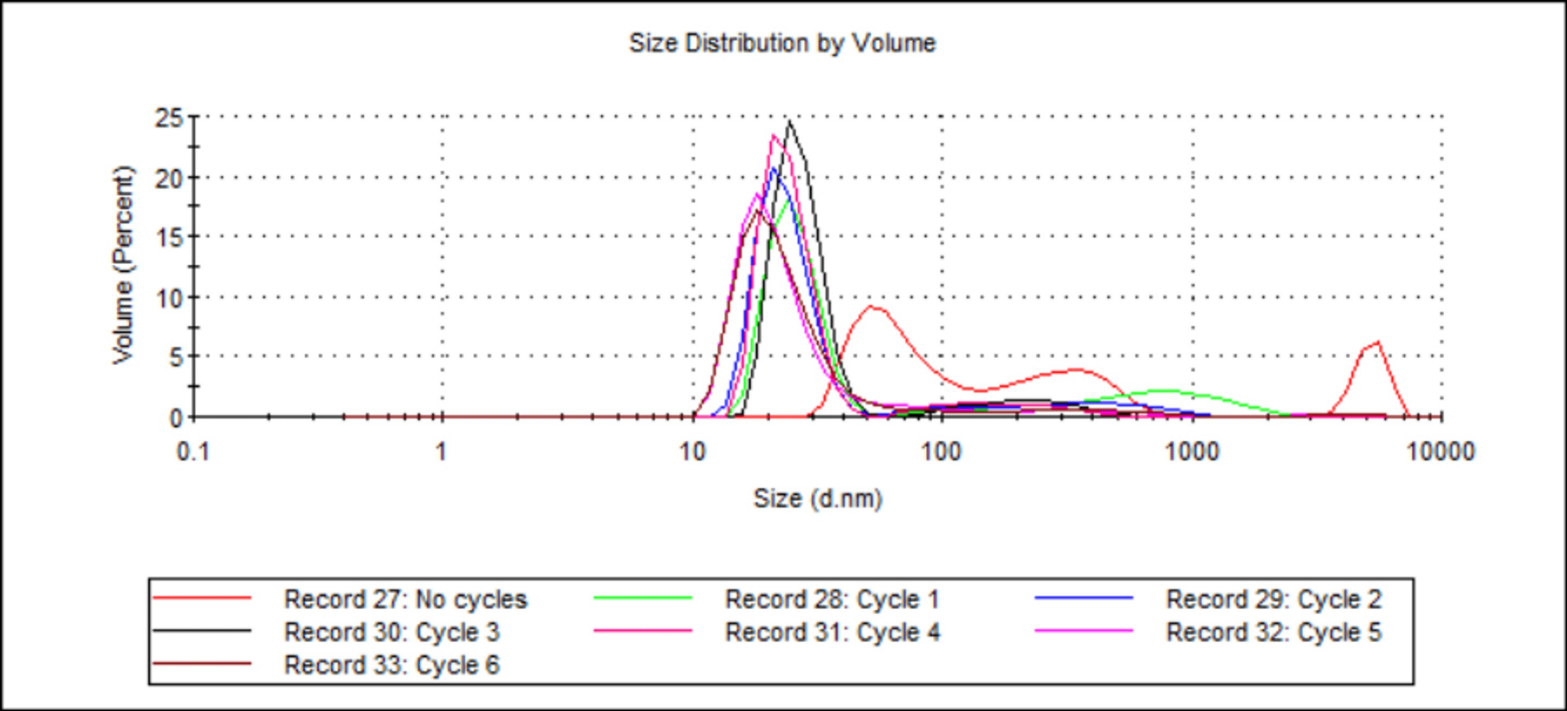
Volume-weighted particle size distributions showing the effect of number of heating and cooling cycles on 888 SLN sizes. For each group, n = 3. This figure illustrates the evolution of particle size distributions for 888 SLNs after successive heating and cooling cycles (ranging from no cycles to six cycles). The x-axis represents the particle size in nanometres (d.nm) on a logarithmic scale, and the y-axis represents the volume percentage of particles at a given size. Initially (Record 27, red line, "no cycles"), the formulation exhibits a broad distribution with a significant presence of larger particles extending into the micron range. Following the first cycle (Record 28, green line, "Cycle 1"), there is a dramatic shift, with a prominent peak emerging in the nanometre range (around 20-30 nm), though some larger particles remain. Subsequent cycles (Records 29-33, Cycles 2-6) show further refinement, yielding consistently sharp and narrow primary peaks clustered tightly around 20 nm, with a notable reduction in the volume percentage of larger aggregates. 888: Compritol 888 ATO (solid lipid); SLN: solid lipid nanoparticles; d.nm: diameter in nanometres

One formulation was created and underwent six heating and cooling cycles. Size measurements were taken before any cycles and after every cycle (see Table 1 and Table 2; samples 4, 5, 8, 9, 12, 14, and 23). Figure 5 shows the mean D50 values for each sample, while Figure 6 shows the volume-weighted particle size distributions for each sample (derived from Zetasizer Nano software version 8.02).

Effect of Changing the Total Surfactant Ratio on Particle Size

**Figure 7:**
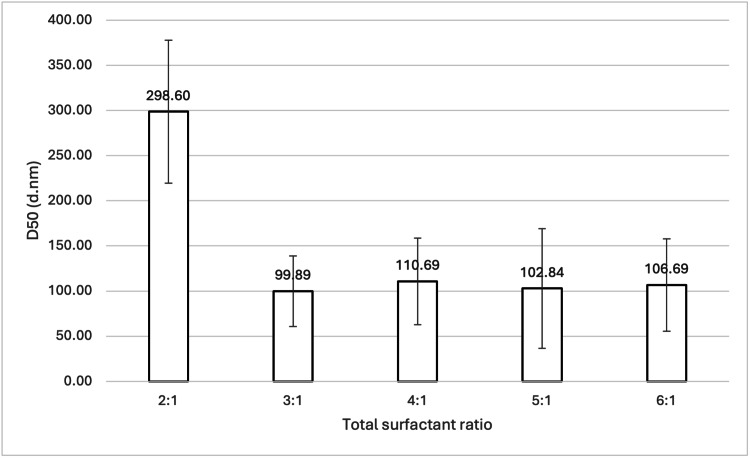
Effect of Tween20-to-monoolein ratio on SLN D50 values. For each group, n = 3. A one-way ANOVA showed that there was no significant difference in the mean D50 values, indicating that the ratio of Tween20 to monoolein did not significantly affect the particle size of SLN samples. 888: Compritol 888 ATO (solid lipid); SLN: solid lipid nanoparticles; D50: percentile value indicating that 50% of all particles in the sample are smaller than the given size; ANOVA: analysis of variance

**Figure 8:**
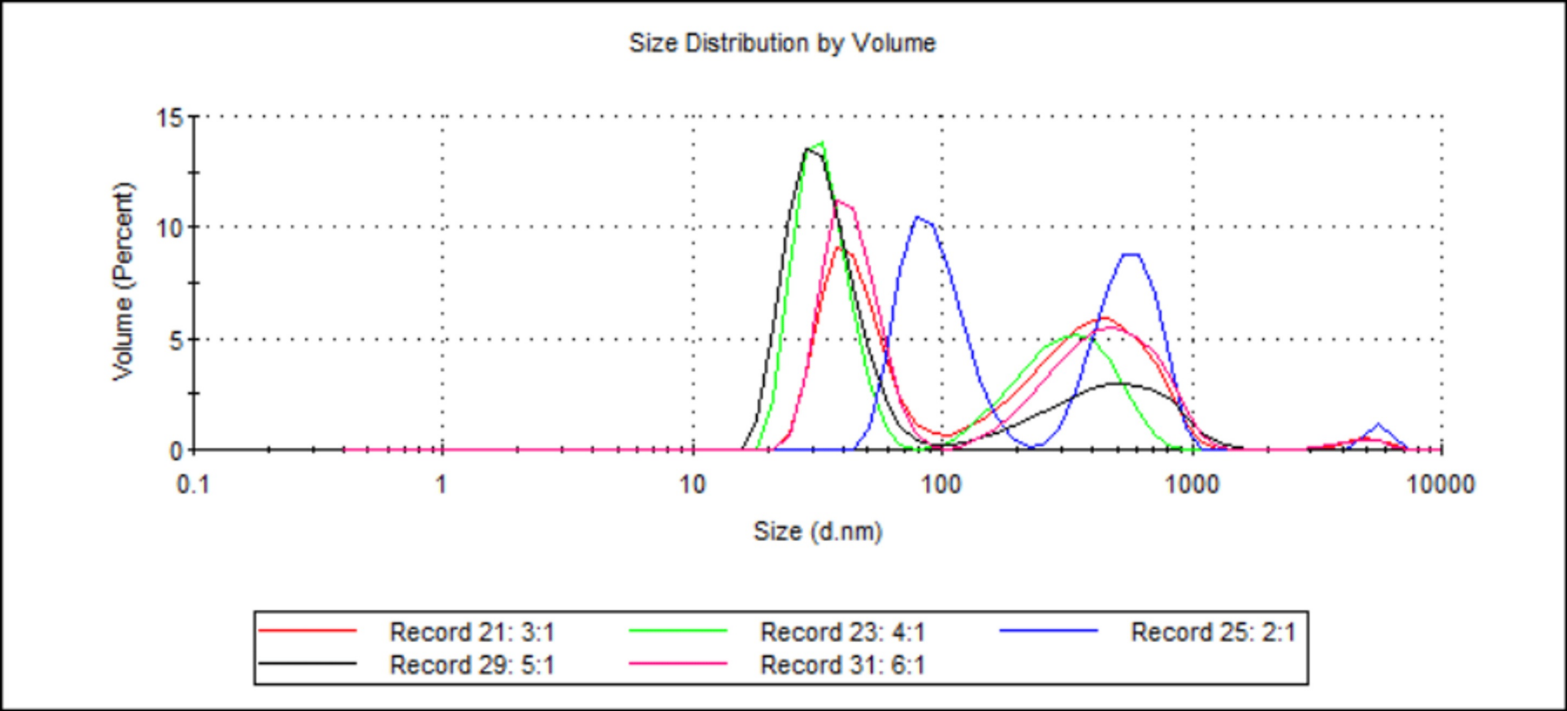
Volume-weighted particle size distributions illustrating the effect of Tween20-to-monoolein ratio on 888 SLN sizes. For each group, n = 3. This figure displays the volume-weighted particle size distribution profiles for 888 SLN formulations prepared with varying ratios of Tween20 to monoolein (2:1, 3:1, 4:1, 5:1, and 6:1). The x-axis represents the particle size in nanometres (d.nm) on a logarithmic scale, and the y-axis represents the volume percentage of particles at a given size. All formulations predominantly exhibit bimodal distributions, with primary peaks ranging from approximately 30 nm to 80 nm and secondary, broader peaks indicating larger particles or aggregates ranging from 300 nm to over 1000 nm. The 2:1 ratio (Record 25, blue line) shows a significant population of larger particles. Conversely, the 5:1 (Record 29, black line) and 6:1 (Record 31, pink line) ratios display primary peaks that are slightly smaller and appear to have a reduced volume percentage of these larger secondary populations compared to other ratios. 888: Compritol 888 ATO (solid lipid); SLN: solid lipid nanoparticles; d.nm: diameter in nanometres

Five samples were analysed (see Table 1 and Table 2; samples 19, 20, 21, 22, and 24), with the ratio between Tween20 and monoolein being the sole difference between them. Each sample's ratio of Tween20 to monoolein was different, being either 2:1, 3:1, 4:1, 5:1, or 6:1. Figure 7 shows the D50 values for each sample, while Figure 8 shows the volume-weighted particle size distributions for each sample (derived from Zetasizer Nano software version 8.02). 

Effect of Different Water-Soluble Surfactants on Particle Size

**Figure 9:**
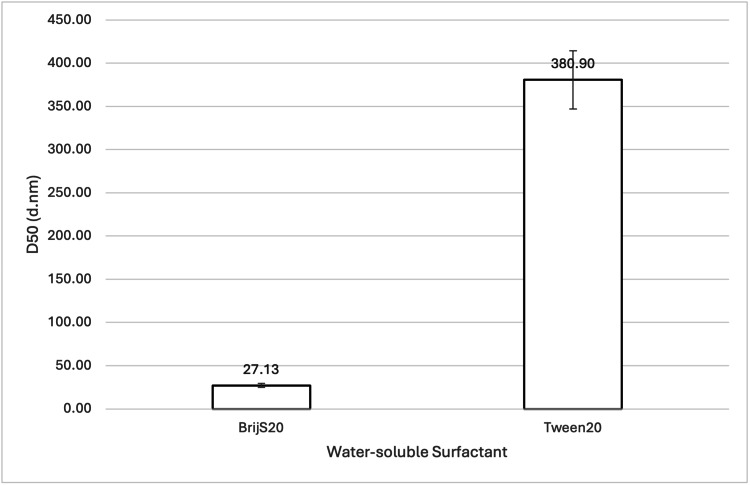
Effect of BrijS20 and Tween20 on 888 SLN D50 values. For each group, n = 3. A one-way ANOVA showed a significant difference in the mean D50 values at a 95% confidence interval (p = 0.0008186), indicating that BrijS20 led to significantly smaller 888 SLN particle sizes compared to Tween20. BrijS20: water-soluble surfactant; Tween20: water-soluble surfactant; 888: Compritol 888 ATO (solid lipid); SLN: solid lipid nanoparticles; D50: percentile value indicating that 50% of all particles in the sample are smaller than the given size; ANOVA: analysis of variance

**Figure 10:**
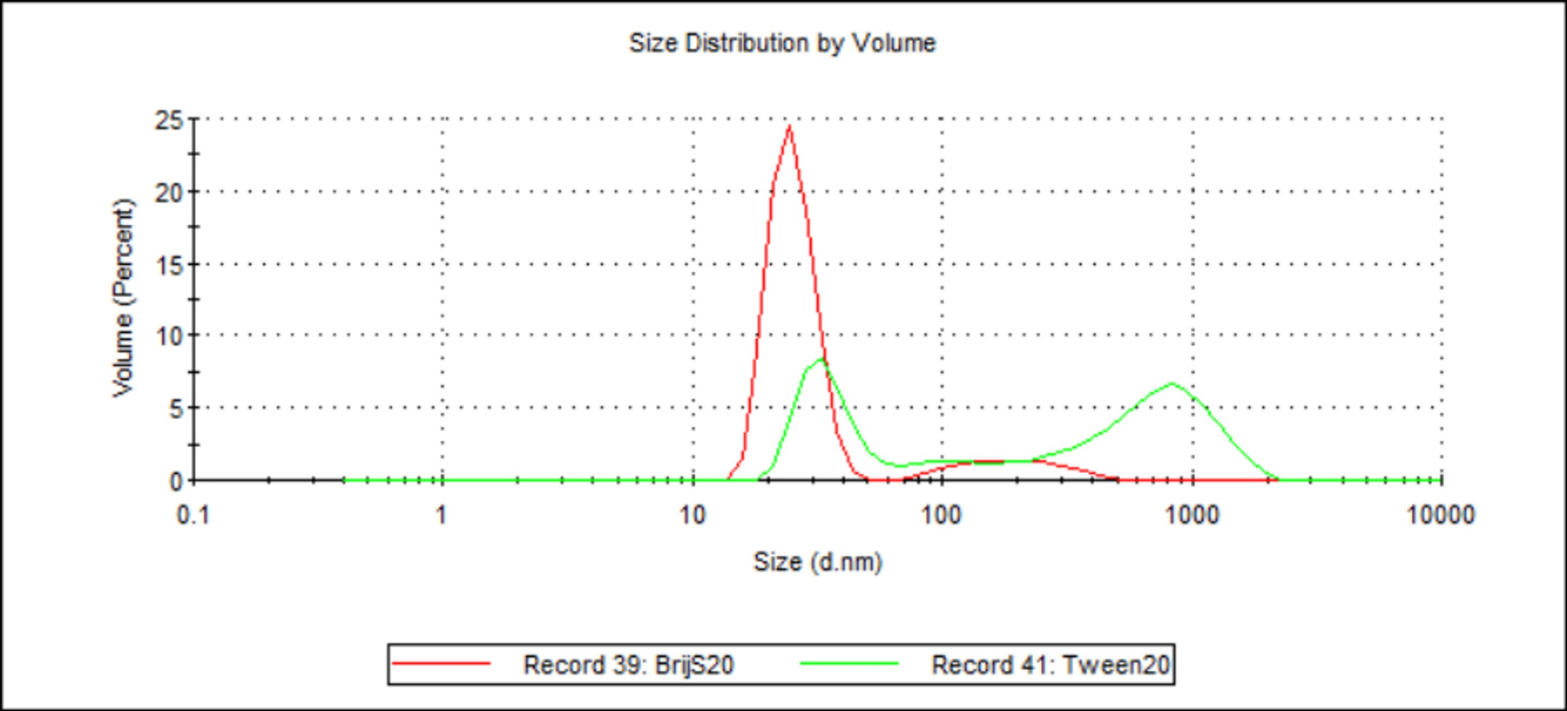
Volume-weighted particle size distributions illustrating the effect of different water-soluble surfactants on 888 SLN sizes. For each group, n = 3. This figure compares the volume-weighted particle size distribution profiles for 888 SLN formulations prepared with either BrijS20 (Record 39, red line) or Tween20 (Record 41, green line) as the water-soluble surfactant. The x-axis represents the particle size in nanometres (d.nm) on a logarithmic scale, and the y-axis represents the volume percentage of particles at a given size. The BrijS20-formulated SLNs exhibit a remarkably sharp and narrow primary peak centred around 15-20 nm and a small secondary peak around 200 nm. In contrast, the Tween20-formulated SLNs show a significantly broader and more polydisperse distribution, characterised by a smaller peak around 30-40 nm and a much more prominent, broad peak in the micron range (around 800-1000 nm), with smaller intermediate populations. This visually suggests that BrijS20 yields smaller and more uniform nanoparticles compared to Tween20. 888: Compritol 888 ATO (solid lipid); SLN: solid lipid nanoparticles; d.nm: diameter in nanometres

Two samples (see Table 1 and Table 2; samples 12 and 25) were analysed, with the choice of water-soluble surfactant being the sole difference between them. The samples contained either BrijS20 or Tween20. Figure 9 shows the mean D50 values for each sample, while Figure 10 shows the volume-weighted particle size distributions for each sample (derived from Zetasizer Nano software version 8.02).

ATO5 cold bursting

The following results are for SLN formulations where ATO5 was used as the lipid. 

Effect of Changing the Total Surfactant Concentration on Particle Size

**Figure 11:**
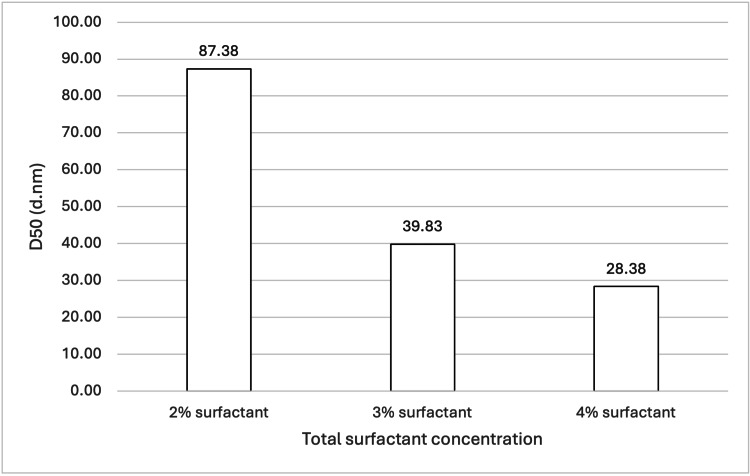
Effect of total surfactant concentration on ATO5 SLN D50 values. For each group, n = 3. A one-way ANOVA showed a significant difference between the mean D50 values (p = 0.01772). Tukey's HSD post hoc analysis revealed a significant decrease in SLN particle size at a 95% confidence interval when total surfactant concentration increased from 2 wt% to either 3 wt% (p = 0.04590) or 4 wt% (p = 0.01887). However, no significant difference in particle size was observed between 3 wt% and 4 wt% total surfactant concentrations. ATO5: Precirol ATO 5 (solid lipid); SLN: solid lipid nanoparticles; D50: percentile value indicating that 50% of all particles in the sample are smaller than the given size; ANOVA: analysis of variance; HSD: honestly significant difference

**Figure 12:**
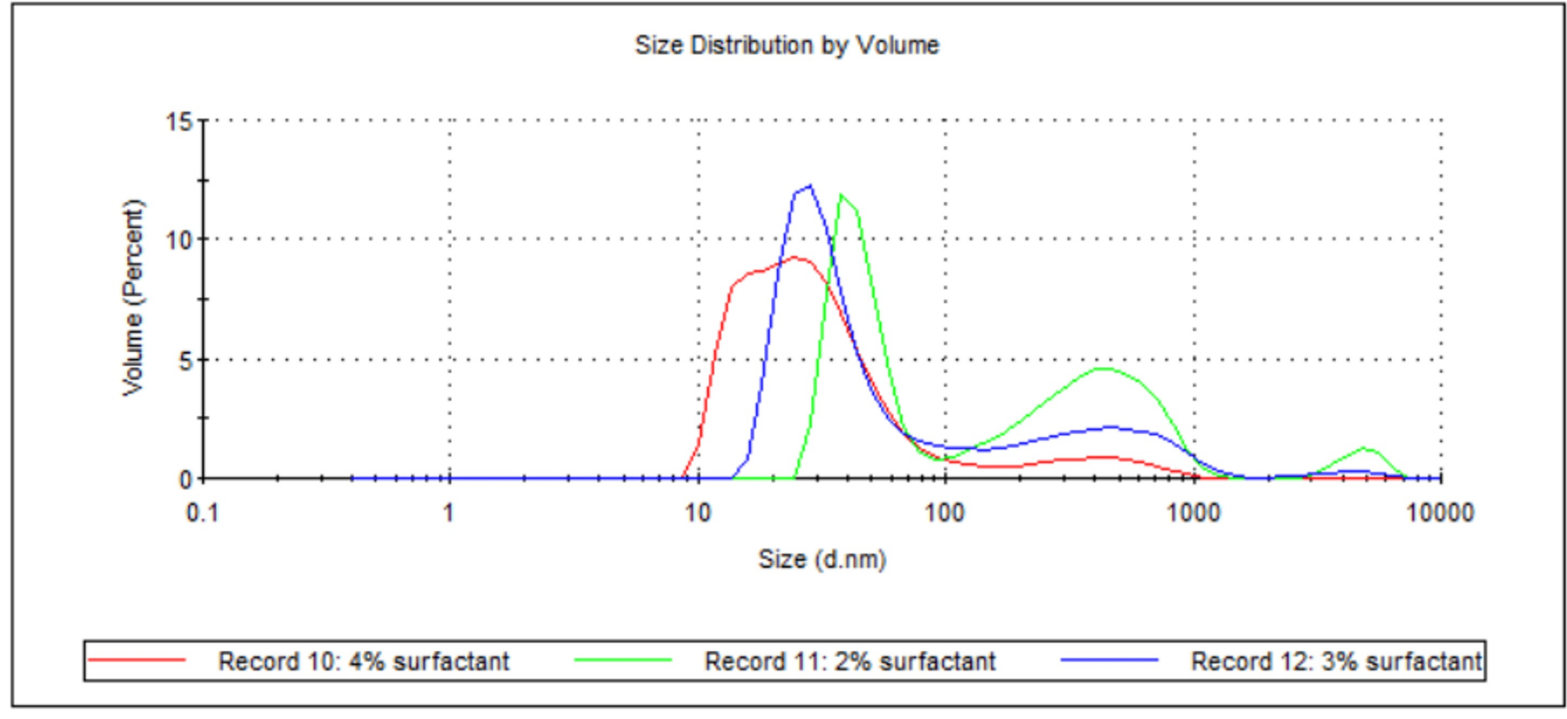
Volume-weighted particle size distributions showing the effect of total surfactant concentration on ATO5 SLN sizes. For each group, n = 3. This figure illustrates the volume-weighted particle size distribution profiles for ATO5 SLN formulations prepared with varying total surfactant concentrations (2 wt%, 3 wt%, and 4 wt%). The x-axis represents the particle size in nanometres (d.nm) on a logarithmic scale, and the y-axis represents the volume percentage of particles at a given size. The 4 wt% surfactant formulation (Record 10, red line) exhibits the smallest primary peak, centred around 15-20 nm, with minimal populations of larger particles. In contrast, the 2 wt% (Record 11, green line) and 3 wt% (Record 12, blue line) formulations show broader distributions with primary peaks at slightly larger sizes (20-40 nm) and more pronounced secondary populations of larger particles, particularly around 500-700 nm for the 2 wt% formulation. This visually suggests that increasing total surfactant concentration improves particle size reduction and homogeneity for ATO5 SLNs. ATO5: Precirol ATO 5 (solid lipid); SLN: solid lipid nanoparticles; d.nm: diameter in nanometres

Three samples (see Table 1 and Table 2; samples 13, 17, and 18) were analysed, with the total surfactant concentration being the sole difference between them. Each sample contained either 2 wt%, 3 wt%, or 4 wt% (with respect to water) total surfactant concentration. Figure 11 shows the mean D50 values for each sample, while Figure 12 shows the volume-weighted particle size distributions for each sample (derived from Zetasizer Nano software version 8.02).

Effect of Changing the Total DTX Concentration on Particle Size

**Figure 13:**
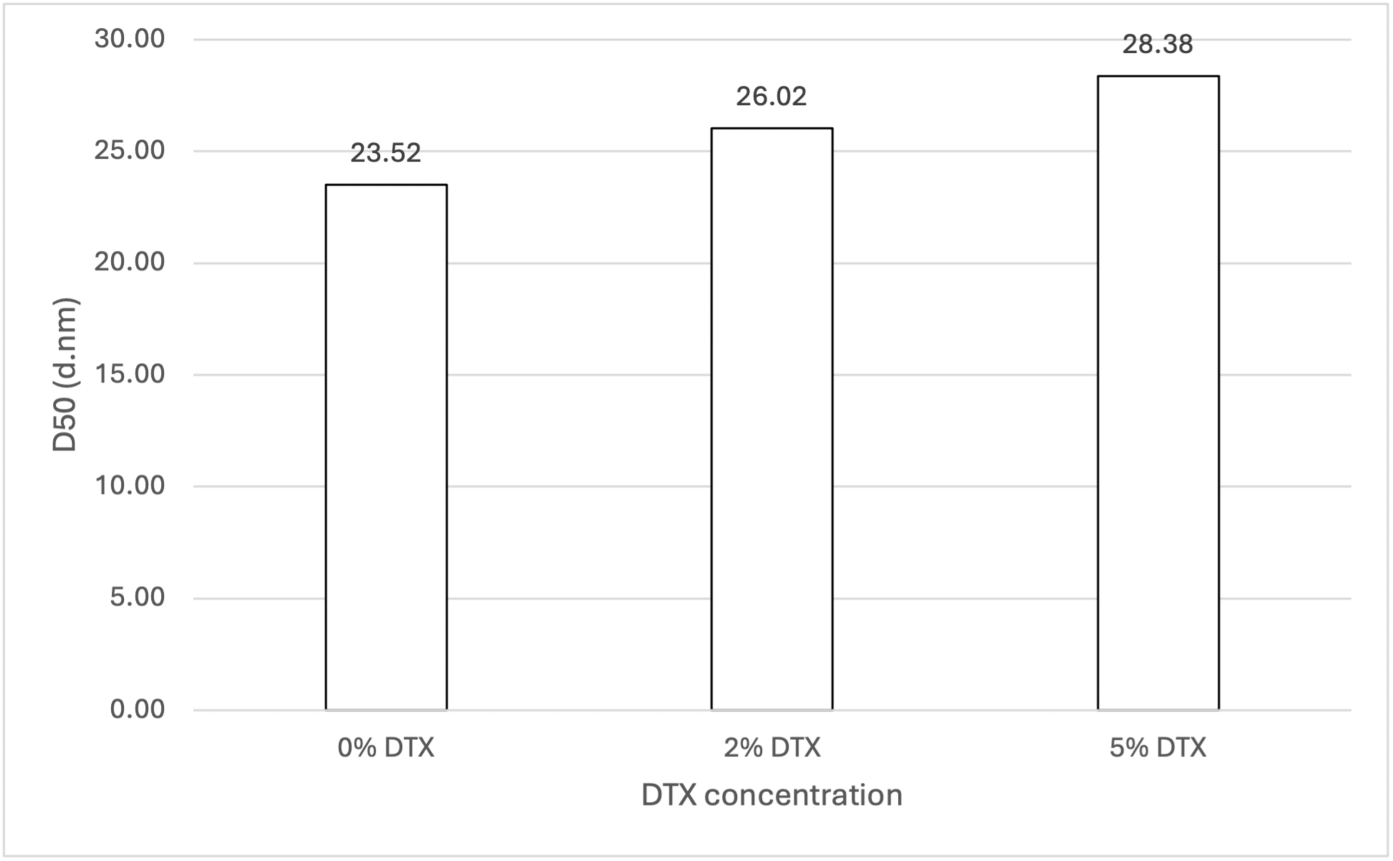
Effect of DTX concentration on ATO5 SLN D50 values. For each group, n = 3. A one-way ANOVA showed that there was no significant difference in the D50 values, indicating that varying DTX concentrations (0 wt%, 2 wt%, and 5 wt% with respect to ATO5) did not significantly affect the particle size of ATO5 SLN samples. DTX: docetaxel; ATO5: Precirol ATO 5 (solid lipid); SLN: solid lipid nanoparticles; ANOVA: analysis of variance; D50: percentile value indicating that 50% of all particles in the sample are smaller than the given size

**Figure 14:**
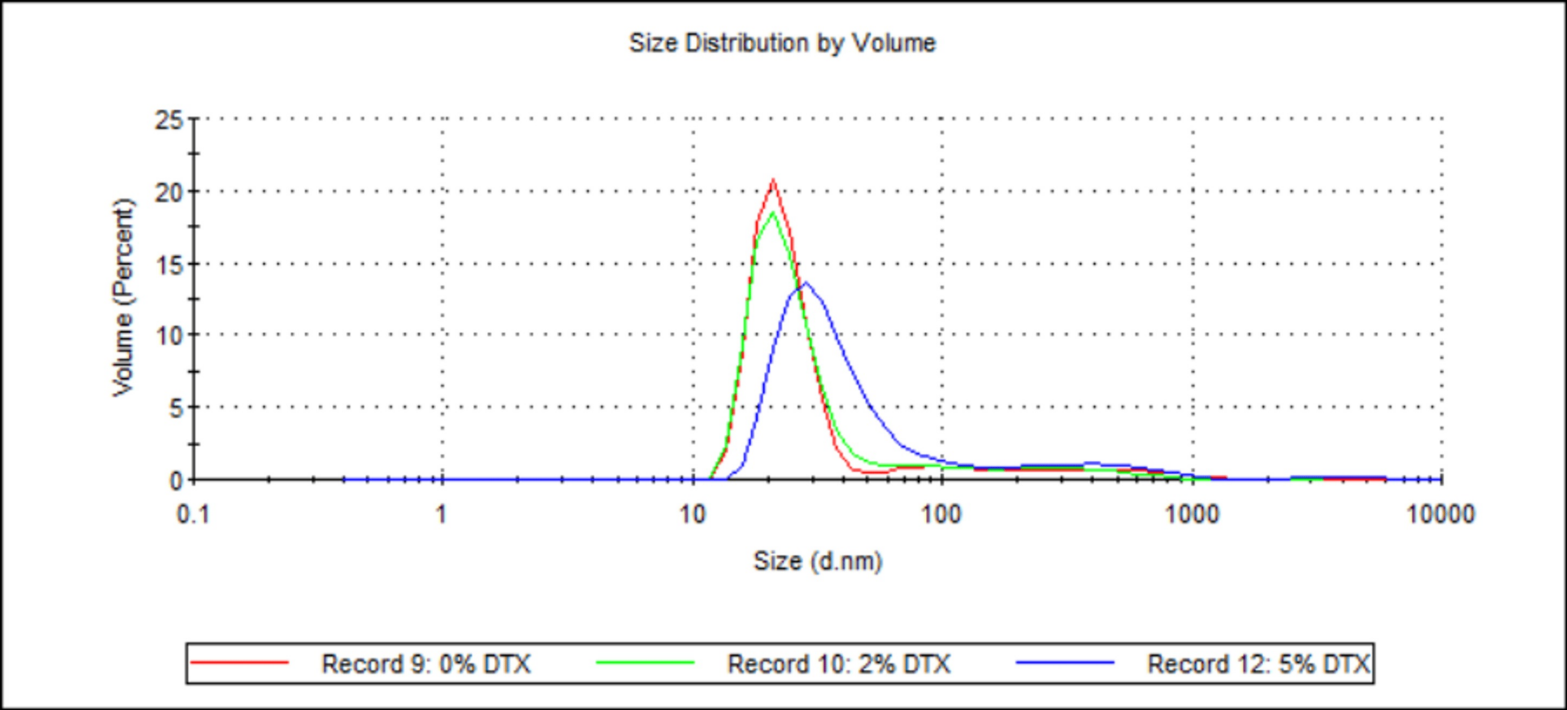
Volume-weighted particle size distributions showing the effect of DTX concentration on ATO5 SLN sizes. For each group, n = 3. This figure illustrates the volume-weighted particle size distribution profiles for ATO5 SLN formulations with varying DTX concentrations (0 wt%, 2 wt%, and 5 wt% with respect to ATO5). The x-axis represents the particle size in nanometres (d.nm) on a logarithmic scale, and the y-axis represents the volume percentage of particles at a given size. All three formulations display prominent and relatively narrow primary peaks, demonstrating the successful formation of sub-30 nm nanoparticles created with up to 5 wt% DTX. DTX: docetaxel; ATO5: Precirol ATO 5 (solid lipid); SLN: solid lipid nanoparticles; d.nm: diameter in nanometres

Three samples (see Table 1 and Table 2; samples 8, 11, and 13) were analysed, with the total DTX concentration being the sole difference between them. Each sample contained either 0 wt%, 2 wt%, or 5 wt% (with respect to ATO5) DTX. Figure 13 shows the mean D50 values for each sample, while Figure 14 shows the volume-weighted particle size distributions for each sample (derived from Zetasizer Nano software version 8.02).

Effect of Different Water-Soluble Surfactants on Particle Size

**Figure 15:**
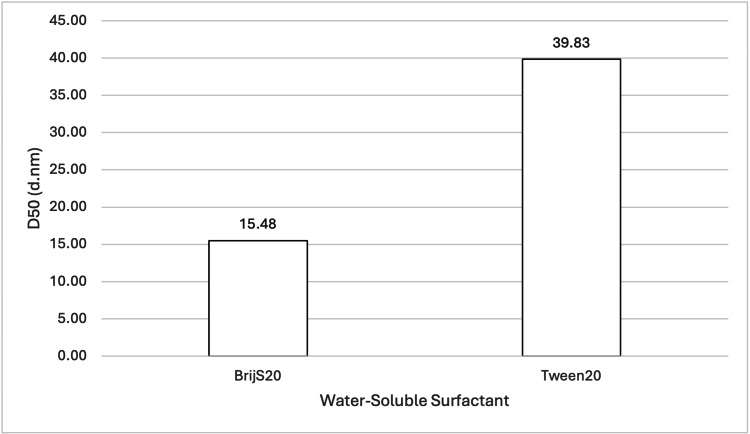
Effect of BrijS20 and Tween20 on ATO5 SLN D50 values. For each group, n = 3. A one-way ANOVA showed a significant difference in the D50 values at a 95% confidence interval (p = 0.001898), indicating that BrijS20 led to significantly smaller ATO5 SLN particle sizes compared to Tween20. BrijS20: water-soluble surfactant; Tween20: water-soluble surfactant; ATO5: Precirol ATO 5 (solid lipid); SLN: solid lipid nanoparticles; D50: percentile value indicating that 50% of all particles in the sample are smaller than the given size; ANOVA: analysis of variance

**Figure 16:**
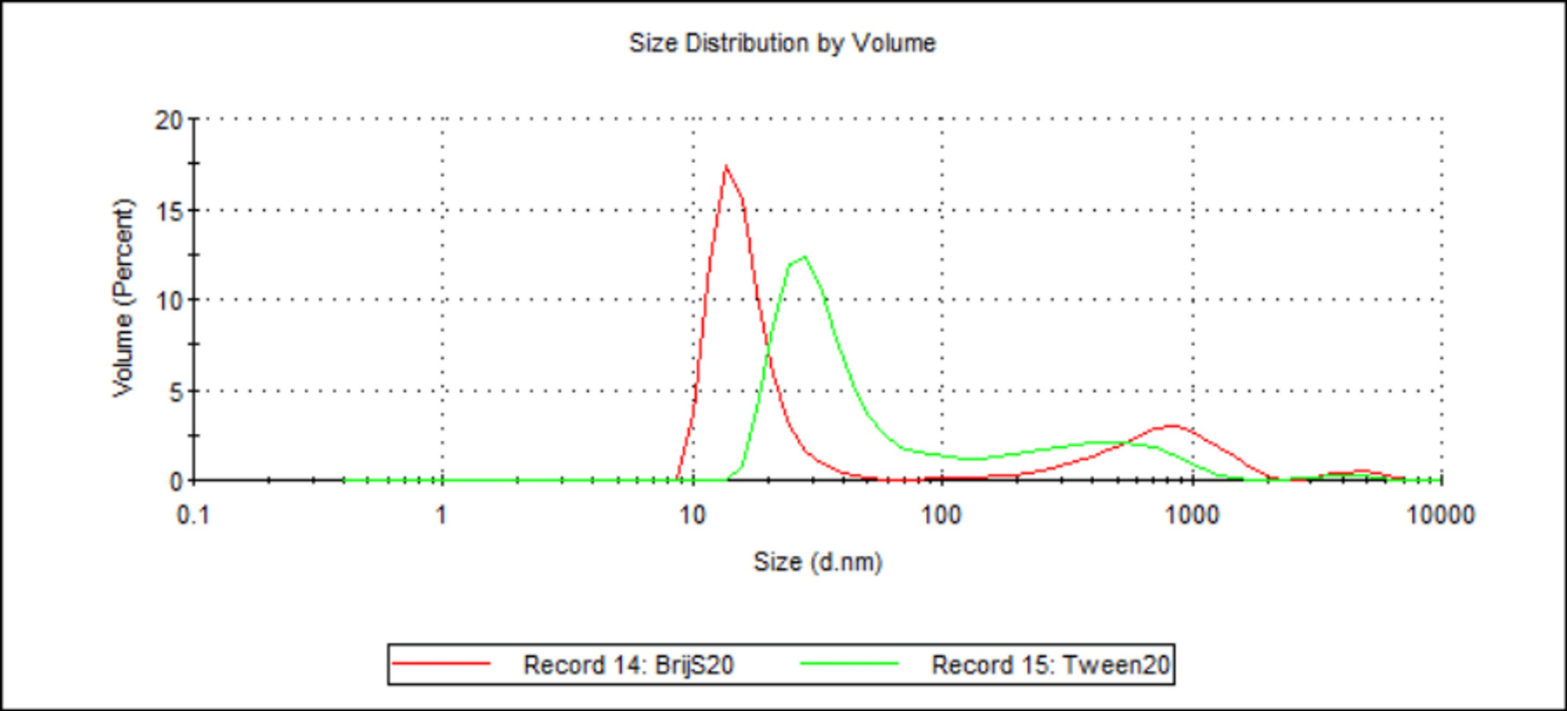
Volume-weighted particle size distributions illustrating the effect of different water-soluble surfactants on ATO5 SLN sizes. For each group, n = 3. This figure compares the volume-weighted particle size distribution profiles for ATO5 SLN formulations prepared with either BrijS20 (Record 14, red line) or Tween20 (Record 15, green line) as the water-soluble surfactant. The x-axis represents the particle size in nanometres (d.nm) on a logarithmic scale, and the y-axis represents the volume percentage of particles at a given size. The BrijS20-formulated SLNs exhibit a sharp and narrow primary peak, centred around 10-15 nm, with a secondary peak in the micron range (800-1000 nm). Tween20-formulated SLNs show a similar bimodal distribution, with the primary peak around 30-40 nm and the secondary peak around 500-800 nm. Visually, this graph suggests that BrijS20 creates a higher proportion of sub-30 nm ATO5 SLNs when compared to Tween20. ATO5: Precirol ATO 5 (solid lipid); SLN: solid lipid nanoparticles; d.nm: diameter in nanometres; DTX: docetaxel

Two samples (see Table 1 and Table 2; samples 1 and 16) were analysed, with different water-soluble surfactants being the sole difference between them. Each sample contained either BrijS20 or Tween20 as its water-soluble surfactant. Figure 15 shows the mean D50 values for each sample, while Figure 16 shows the volume-weighted particle size distributions for each sample (derived from Zetasizer Nano software version 8.02).

Summary of Key Findings

Our systematic investigation of cold-burst parameters for SLN production yielded several key findings regarding particle size optimisation:

Sub-30 nm SLN formation: Stable DTX-loaded SLNs with mean D50​ values below 30 nm were consistently achieved for formulations using both 888 and ATO5 as lipid components.

Impact of surfactant concentration: A significant decrease in particle size was observed with increasing total surfactant concentration, with the smallest sub-30 nm particles forming at 4 wt% total surfactant for both lipid types.

Surfactant type superiority: BrijS20 consistently facilitated the creation of significantly smaller nanoparticles compared to Tween20 across both lipid types, demonstrating its superior efficacy in particle size reduction within the cold-burst method.

Effect of DTX loading: DTX loading up to 5 wt% (with respect to lipid, equating to 5 mg) did not significantly affect the particle size of the resulting SLNs for either lipid, suggesting its potential for incorporation without compromising the desired small size.

Cycling efficiency: The most pronounced size reduction for 888 SLNs occurred within the first heating and cooling cycle, with subsequent cycles yielding only minor further decreases in particle size.

Tween20-to-monoolein ratio: While varying the Tween20-to-monoolein ratio for 888 SLNs allowed cold bursting to occur, formulations with Tween20 did not achieve sub-30 nm particle sizes.

## Discussion

This study successfully demonstrated the ability of the novel cold-burst method to produce SLNs that achieved sub-30 nm sizes using both 888 and ATO5 as lipid components. As mentioned previously, smaller nanoparticles allow nanoparticles to passively target tumours through the EPR effect [13], and sub-30 nm nanoparticles may distribute more deeply into tumour tissues [14]. Research has also indicated that nanoparticles within the 25-50 nm range are more efficiently taken up by cells via endocytosis pathways [14] and that larger (>200 nm) nanoparticles risk being filtered by the liver or causing embolism [19]. In addition to the clinical benefits of sub-30 nm nanoparticles, maintaining a small particle size may contribute significantly to the physical stability of the nanocarrier system. Smaller and more narrowly distributed nanoparticles may exhibit better stability and be less prone to aggregation. This is crucial for maintaining a homogenous formulation for storage and therefore potentially ensuring consistent drug delivery characteristics in vivo. The consistent achievement of nanoparticles <30 nm is of paramount importance for optimising nanocarrier function and is expected to enhance the therapeutic efficacy of encapsulated drugs like DTX in cancer treatment.

Statistical analysis of this study's data showed similar trends in 888 and ATO5 cold bursting in terms of how certain variables affect particle size.

Both 888 and ATO5 cold bursting demonstrated significant decreases in mean D50 values when total surfactant concentration was increased from 2 wt% to 3 or 4 wt% (see Figure 1 and Figure 11). For both lipids, 4 wt% total surfactant created the smallest sub-30 nm particles. Previous literature has shown the effect that added surfactant can have on both particle size and the stability of nanosized materials. In terms of particle size, higher surfactant concentrations stabilise newly developed hydrophobic surfaces and decrease surface tension [20]. This is relevant for this study because cold bursting results in larger particles "bursting" to create many smaller nanoparticles, which contain hydrophobic lipid surfaces. Having enough surfactant in solution to sufficiently cover the hydrophobic surface of the SLNs may reduce particle aggregation and therefore lead to a lower particle size [21]. In the case of 2 wt% surfactant, the lowered surfactant concentration may have resulted in surfactant molecules not adsorbing quickly enough relative to the rate of particle-particle collision, resulting in aggregation and a larger D50 value. Further insights into our report's findings are provided by the volume-weighted particle size distributions (Figure 2 and Figure 12). For instance, Figure 2 illustrates how the 3 wt% and 4 wt% surfactant formulations for 888 SLNs yielded distinctly narrower and sharper primary peaks, centred around 15-20 nm. This suggests a superior homogeneity compared to the 2 wt% formulation, which exhibited a broader peak and a more pronounced tail of larger particles. Similarly, Figure 12 demonstrates that for ATO5 SLNs, increasing the surfactant to 4 wt% resulted in the sharpest and most homogenous distribution, with significantly reduced populations of larger aggregates compared to the lower surfactant concentrations. These visual characteristics align with the importance of having sufficient surfactant to prevent particle aggregation. The literature states that a cold burst has not occurred when surfactant concentrations are too large (around 6 wt%) [11]. In this study, no results were observed that showed a decrease in the ability for cold bursts to occur with increased surfactant concentrations. Only three total surfactant concentrations were explored (2%, 3%, and 4%). More research into a wider range of total surfactant concentrations may be necessary to truly optimise creation of sub-30 nm particles via cold burst.

For both 888 and ATO5, statistical analysis showed there was no significant difference between the D50 values for 0 wt%, 2 wt%, and 5 wt% DTX (see Figure 3 and Figure 13). However, with ATO5, there was a consistent trend observed of D50 values increasing as DTX concentration increases. With ATO5, increasing DTX concentration from 0 to 2 to 5 wt% led to an increase in D50 values from 23.52 to 26.02 to 28.38, respectively. The volume-weighted size distribution graphs (Figure 4 and Figure 14) offer additional visual context to these statistical findings. Figure 4 shows that while the mean particle sizes for 0 wt%, 2 wt%, and 5 wt% DTX were not significantly different, 0 wt% DTX had the smallest and narrowest peak. Similarly, Figure 14 illustrates that although all concentrations remained sub-30 nm, the primary peak for the 5 wt% DTX formulation was marginally broader and shifted to a slightly larger size compared to the unloaded formulation. These observations provide a more nuanced understanding of how DTX loading, even without a statistically significant impact on the mean size, may affect the overall size distribution. This trend is observed in the literature that explores alternative ways of producing SLNs. Ketoprofen-loaded SLNs that were created by ultrasonication saw a particle diameter size increase from 175 nm to 244 nm as ketoprofen was increased from 2 wt% to 6 wt% [21]. The same increase is seen widely across different research papers and is thought to be due to an increased solid content when preparing initial emulsions, which causes an increased size of primary emulsions and therefore an increased size of final nanoparticles [22]. This could explain why ATO5 sizes increased as DTX concentration increased. For 888, no consistent trend was seen with an increased DTX concentration. This was surprising due to the trends that have been seen in the literature and could potentially be due to the low number of Zetasizer readings used to create the mean D50 values. Only three Zetasizer readings were used to create mean D50 values, so potentially having more Zetasizer readings could improve the reproducibility of results, which might lead to results that align with the literature. Furthermore, creating multiple samples with the same material composition and using readings from each sample to create the mean D50 values would reduce the effect of any errors which may arise in the cold-burst method. This could further improve the reproducibility of results, but due to time constraints, it was not possible to create multiple samples for each formulation. In this study, the maximum amount of DTX used was 5 mg (5 wt%). Research has shown that SLNs loaded with up to 2 mg of DTX showed cytotoxicity to prostate cancer cells, while unloaded SLNs showed no cytotoxicity to the same cells [23]. This would suggest, based on the literature, that the 2 mg (2 wt%) and 5 mg (5 wt%) DTX used in this study are sufficiently large enough to be effective against prostate cancer cells. Furthermore, this study's results showing that there are no significant differences in the sizes of 0 wt%, 2 wt%, and 5 wt% DTX-loaded SLNs suggest that enough DTX can be loaded into small-sized SLNs without compromising size and therefore their potential for therapeutic function. This is particularly encouraging as it suggests that cold burst could be used to create sub-30 nm SLNs loaded with a clinically relevant dose of DTX. However, this is assuming that there was a good entrapment efficiency of DTX within these SLNs. One limitation of this study is that drug loading was not quantified, meaning that there is potential that some of the DTX did not remain in the SLNs. Since entrapment efficiency was not specifically explored in this study, more research is needed to confirm how much of the DTX is within the cold-burst-produced SLNs.

When comparing the effect of different water-soluble surfactants on particle size, both ATO5 and 888 showed a significant decrease in D50 values when made with BrijS20 in comparison to Tween20 (see Figure 9 and Figure 15). This significant difference is visually illustrated in the volume-weighted particle size distributions (Figure 10 and Figure 16). In both figures, the BrijS20-formulated SLNs exhibit a sharp and narrow primary peak centred around 15-20 nm, with smaller secondary peaks around 200 nm (888 SLNs) and 1000 nm (ATO5 SLNs). In contrast, the Tween20-formulated SLNs show a larger and more polydisperse distribution, characterised by a primary peak around 30-50 nm and secondary peaks around the 800-1000 nm range that are larger than the secondary peaks seen in the BrijS20 SLNs. This visual evidence suggests that under the process conditions tested, BrijS20 is superior in facilitating the cold-burst process to create sub-<30 nm SLNs for both lipids. In addition to this, Table 1 shows that out of the 13 formulations that created mean D50 values under 30 nm, the smallest seven were all BrijS20-formulated and 10 out of the 13 were BrijS20-formulated. These results suggest that BrijS20 creates smaller and more uniform SLN formulations than Tween20. Just like total surfactant concentration, choice of surfactant may affect SLN's stability and particle size [24]. Research has indicated that 888 and ATO5 SLNs can have their size significantly affected by a change in water-soluble surfactant. 888 and ATO5 SLNs had their particle size increased from 206.9 nm and 276.9 nm to 596.7 nm and 684.5 nm, respectively, when the surfactant was changed from Tween80 to polyvinyl alcohol [25]. A change in particle size due to different surfactants is commonly seen in the literature, which aligns with the results of this study. Research into cold-burst optimisation compared the cold-burst efficiency of Tween20 with monoolein against BrijS20 with monoolein. Tween20 with monoolein was given a cold-burst score of 4.5/5, while BrijS20 with monoolein was given a score of 2.5/5 [11]. A higher cold-burst score indicated that more droplets "burst" into smaller particles, so this study's results disagree with the literature here by suggesting that BrijS20 creates smaller-sized particles. This study used two non-ionic surfactants to create the SLNs, but from the literature, it is known that ionic surfactants may adsorb faster than non-ionic ones [11]. Perhaps research into creating DTX-loaded SLNs by cold burst using ionic surfactants would give more indication into particle size optimisation and offer more options for formulations that result in sub-30 nm SLNs. While BrijS20 consistently yielded significantly smaller nanoparticles across both lipid types compared to Tween20, the choice of surfactant for therapeutic applications must also consider biocompatibility. Future comprehensive safety evaluations, including cytotoxicity testing, are essential to determine the most clinically viable surfactant given that variations in surfactant type can lead to different cellular responses despite achieving desired particle sizes.

The effect of cycling 888 SLNs multiple times was investigated in this study, and results showed that the only significant difference in particle size was seen after one cycle. One heating and cooling cycle led to a mean D50 size reduction of 191.50 nm to 31.18 nm (see Figure 5). The mean D50 further reduced to 21.04 nm after six cycles, but a Tukey's HSD post hoc analysis deemed there to be no significant difference. These results show that for 888, the largest size reduction is seen within the first heating and cooling cycle. Although size reduction continues to occur with more cycles, the reduction is very small in comparison to the effect of the first cycle. The dramatic effect of the first heating and cooling cycle is visually striking in the volume-weighted particle size distribution graph (Figure 6). The initial formulation displays a very broad distribution with a significant presence of larger particles. After just one cycle, the distribution shifts drastically, with a prominent peak emerging in the nanometre range. While subsequent cycles (2-6) result in minor changes to the mean size, the distributions show a continuous refinement of the particle population, with the gradual reduction of the smaller peaks representing larger aggregates. This is visual evidence that suggests the first cycle is the most impactful, while subsequent cycles serve to refine the distribution and improve homogeneity. Our results align with the literature that demonstrates that the first heating and cooling cycle led to a size decrease of lipid particles from 100 μm to 0.4 μm [11]. The literature suggests that altering the heating and cooling rates during heating and cooling cycles may improve the cold-burst mechanism. Slower cooling rates result in larger nanoparticles after heating, and a slower heating rate allows more time for the aqueous phase to penetrate the particles and cause a cold burst [10]. Similarly to the literature, this study used a heating rate of 0.5°C/minute. However, in this study, the samples were rapidly cooled in an ice bath for 30 seconds where the cooling rate was variable from 2 to 4°C/s. The literature cooled samples at a fixed rate of 2.5°C/s [11]. Future research into reducing the sizes of 888 SLNs should explore slower heating rates, alongside more precise and controlled cooling rates. One paper showed that it is possible to achieve >99% of particles around 20 nm after four cycles using ATO5 [11]. One limitation of this study was that only D50 was measured. Although it is highly important to prove that sub-30 nm DTX-loaded SLNs can be created by cold burst, it is also important to show that the method is energy-efficient and has a scalable production process. If every formulation needs to undergo rigorous filtering or processing to isolate these sub-30 nm SLNs, large-scale production may not be practical. Future research into creating drug-loaded SLN formulations of a homogenous size or a narrow size distribution that are also sufficiently small would be important in showing that cold burst is viable for larger-scale production.

This study's results showed that for 888 SLNs, no significant difference was seen in D50 values when the ratio of Tween20 to monoolein was changed (see Figure 7). A ratio of 2:1 created the largest particles, with a D50 of 298.60 nm, and all other samples had D50s around 100 nm. For all samples created with 888 and Tween20, no formulation had a mean D50 <30 nm. This suggests that the combination of 888 and Tween20 would not be useful in creating DTX-loaded SLNs for cancer therapy. The volume-weighted particle size distributions (Figure 8) provide visual detail behind these findings. The graph clearly shows that most of the formulations, particularly the 2:1 ratio, exhibit a bimodal distribution with a significant population of large particles or aggregates beyond 100 nm. This contrasts sharply with the narrow, monomodal distributions observed in successful sub-30 nm formulations and visually highlights how these formulations were unable to achieve the desired small particle sizes, despite not showing a significant difference in mean D50 values across the ratios. The presence of these larger populations underlines the importance of a suitable surfactant ratio for effective cold bursting and suggests that the combination of 888 and Tween20 may be less effective in producing the desired monomodal, sub-30 nm SLNs. The literature explores the effect of different surfactant ratios using Tween20 and monoolein. Research indicates that cold bursting efficiency decreased for ratios of 4:1 and 5:1 and cold bursting was nearly fully suppressed at a 6:1 ratio. In addition, ratios of 2:1 and 3:1 resulted in the most efficient cold bursting [11]. Although the literature used coconut oil as opposed to 888, our results differ significantly. This may be explained by potential confounding factors that were not explored in this experiment. Literature suggests that to achieve efficient cold bursting, the three-phase contact angle at the frozen oil-water-air contact line should be ≤30° and that at contact angles of around 100°, water-in-oil-in-water drops were formed during initial emulsion formation. For our study, this may hinder the ability for DTX to be loaded into the lipid droplets since the lipid droplets would contain water and DTX is highly hydrophobic [26]. The literature also details how increasing temperature can lead to a smaller contact angle when using Tween20 and monoolein with coconut oil [10]. Due to 888's different melting range to coconut oil, temperatures that were different from the previous literature were used in this study. The temperature's effect on contact angles and contact angles' effect on cold burst efficiency may explain why this study's results relating to surfactant ratios were different from the literature. In the future, research into contact angles and their effect on DTX-loaded SLNs made by cold burst may be necessary to optimise particle size, potentially allowing for formulations containing 888 and Tween20 to create sub-30 nm SLNs.

Limitations

This study provides valuable insights into the particle size optimisation of DTX-loaded SLNs using the cold-burst method. However, it is important to acknowledge certain limitations that arise from the experimental methodology and the scope of the study.

A key limitation is that drug loading and entrapment efficiency were not quantitatively assessed, meaning the precise amount of DTX encapsulated within the SLNs remains unconfirmed. This study primarily focused on optimising particle size, and while the consistency of size with DTX loading is encouraging, future work is necessary to quantify this aspect. Additionally, the investigations were limited to particle size analysis (D50​) and volume-weighted particle size distribution graphs. Comprehensive characteristics like polydispersity index (PDI), zeta potential, or in vitro drug release kinetics were not measured in this study. These are crucial for understanding stability, sustained drug delivery, and batch homogeneity of nanoparticle formulations. The preclinical nature of this work means that the in vivo efficacy and safety of these cold-burst-derived SLNs in relevant cancer models were not evaluated.

Methodologically, the study explored a limited range of certain parameters, such as total surfactant concentrations (only 2-4 wt%), and relied on a small number of Zetasizer readings (n = 3) per sample, which could affect the reproducibility of some findings and the statistical power to detect subtle effects. This study would be improved if independent batches for each formulation were created in addition to multiple Zetasizer readings. Ideally, >3 batches for each formulation would be created to improve reproducibility. Unfortunately, due to time constraints and material constraints, this was not possible for our study. Furthermore, while efforts were made to standardise the procedure, several methodological aspects could introduce confounding factors or variability. First is the temperature variation during rapid cooling. Although samples were rapidly cooled in an ice bath for 30 seconds at a cooling rate of between 2 and 4°C/s, the exact rate was not fixed. Variations in the ice bath temperature or immersion dynamics could influence particle solidification and final size. This study could be improved by using a fixed-rate cooling system to improve reproducibility and emulate the cooling rate of approximately 2.5°C/s which was seen in prior cold-burst studies [11]. Second is the certain unmeasured physico-chemical parameters. Factors critical to the cold-burst mechanism, such as the three-phase contact angle at the lipid-water-air interface or specific interfacial tension values, were not measured. Variations in these uncharacterised properties across different formulations or batches could also serve as confounding factors influencing the particle size outcomes.

While effective, the ability of the cold-burst method to produce highly homogenous batches with narrow size distributions for large-scale production without rigorous post-processing also requires further investigation, as this was not fully explored in this size optimisation study.

## Conclusions

In this study, the cold-burst method was successfully used to produce <30 nm DTX-loaded SLNs using industrially relevant lipids, 888 and ATO5. This study consistently achieved sub-30 nm particle sizes, particularly with increased total surfactant concentrations (over 2 wt%) and the use of BrijS20 as the water-soluble surfactant. This work suggests the possibility of the cold-burst method as an efficient, low-cost, and scalable technique for producing sub-30 nm DTX-loaded SLNs with tuneable properties, specifically demonstrating its utility for particle size optimisation. The inherent simplicity and energy efficiency of the cold-burst method suggest promising scale-up feasibility for industrial production. However, successful clinical translation of these nanomedicines will also necessitate rigorous adherence to regulatory guidelines governing pharmaceutical manufacturing and nanomedicine development.

Creation of SLN formulations with 2 wt% and 5 wt% DTX showed no significant difference in particle size compared to SLN formulations with 0 wt% DTX. This suggests the potential for high drug loading while maintaining the small nanoparticle size that allows for effective cancer cell targeting. Both lipids showed a significant decrease in particle size when total surfactant concentration was increased, with sub-30 nm nanoparticles observed when surfactant concentration exceeded 2 wt%. In terms of water-soluble surfactants used, BrijS20 created significantly smaller particles than Tween20 for both ATO5 and 888 SLNs. For 888 SLNs, the smallest size reduction was observed during the first heating and cooling cycle, and subsequent cycles after resulted in minor decreases to particle size. Furthermore, changes in the ratio of Tween20 to monoolein for 888 SLNs still allowed cold burst to occur. A 2:1 ratio produced the largest particles, while ratios between 3:1 and 6:1 produced smaller samples. However, as these smaller samples had a D50 of around 100 nm, this suggests that the combination of 888 and Tween20 may not produce sub-30 nm SLNs.

Future work should focus on quantifying the precise drug loading and encapsulation efficiency of DTX within these SLNs. Furthermore, comprehensive in vitro drug release profiles, PDI measurements, cytotoxicity against cancer cell lines, and rigorous in vivo efficacy and safety assessments are essential to validate these cold-burst-derived SLNs as promising and safe therapeutic options for patients with cancer.

## References

[1] Schwarz C, Mehnert W, Lucks JS, Müller RH (1994). Solid lipid nanoparticles (SLN) for controlled drug delivery. I. Production, characterization and sterilization. J Control Release.

[2] Ghasemiyeh P, Mohammadi-Samani S (2018). Solid lipid nanoparticles and nanostructured lipid carriers as novel drug delivery systems: applications, advantages and disadvantages. Res Pharm Sci.

[3] Scioli Montoto S, Muraca G, Ruiz ME (2020). Solid lipid nanoparticles for drug delivery: pharmacological and biopharmaceutical aspects. Front Mol Biosci.

[4] Leslie SW, Soon-Sutton TL, Skelton WP (2024). Prostate cancer. StatPearls [Internet].

[5] (2025). About prostate cancer. https://prostatecanceruk.org/prostate-information/about-prostate-cancer/.

[6] Dong Y, Feng SS (2005). Poly(d,l-lactide-co-glycolide)/montmorillonite nanoparticles for oral delivery of anticancer drugs. Biomaterials.

[7] Chaurasiya A, Singh AK, Jain GK (2012). Dual approach utilizing self microemulsifying technique and novel P-gp inhibitor for effective delivery of taxanes. J Microencapsul.

[8] Cho HJ, Park JW, Yoon IS, Kim DD (2014). Surface-modified solid lipid nanoparticles for oral delivery of docetaxel: enhanced intestinal absorption and lymphatic uptake. Int J Nanomedicine.

[9] Nakamura Y, Mochida A, Choyke PL, Kobayashi H (2016). Nanodrug delivery: is the enhanced permeability and retention effect sufficient for curing cancer?. Bioconjug Chem.

[10] Cholakova D, Glushkova D, Tcholakova S, Denkov N (2020). Nanopore and nanoparticle formation with lipids undergoing polymorphic phase transitions. ACS Nano.

[11] Cholakova D, Glushkova D, Tcholakova S, Denkov N (2021). Cold-burst method for nanoparticle formation with natural triglyceride oils. Langmuir.

[12] Danaei M, Dehghankhold M, Ataei S (2018). Impact of particle size and polydispersity index on the clinical applications of lipidic nanocarrier systems. Pharmaceutics.

[13] Maeda H (2015). Toward a full understanding of the EPR effect in primary and metastatic tumors as well as issues related to its heterogeneity. Adv Drug Deliv Rev.

[14] Sahay G, Alakhova DY, Kabanov AV (2010). Endocytosis of nanomedicines. J Control Release.

[15] Blanco E, Shen H, Ferrari M (2015). Principles of nanoparticle design for overcoming biological barriers to drug delivery. Nat Biotechnol.

[16] Kasongo KW, Pardeike J, Müller RH, Walker RB (2011). Selection and characterization of suitable lipid excipients for use in the manufacture of didanosine-loaded solid lipid nanoparticles and nanostructured lipid carriers. J Pharm Sci.

[17] Baek JS, Cho CW (2015). Comparison of solid lipid nanoparticles for encapsulating paclitaxel or docetaxel. J Pharm Investig.

[18] Gagliardi A, Voci S, Salvatici MC, Fresta M, Cosco D (2021). Brij-stabilized zein nanoparticles as potential drug carriers. Colloids Surf B Biointerfaces.

[19] Kreuter J (2007). Nanoparticles-a historical perspective. Int J Pharm.

[20] McClements DJ (2012). Nanoemulsions versus microemulsions: terminology, differences, and similarities. Soft Matter.

[21] Van Zyl AJ, de Wet-Roos D, Sanderson RD, Klumperman B (2004). The role of surfactant in controlling particle size and stability in the miniemulsion polymerization of polymeric nanocapsules. Eur Polym J.

[22] Kumar R, Singh A, Garg N, Siril PF (2018). Solid lipid nanoparticles for the controlled delivery of poorly water soluble non-steroidal anti-inflammatory drugs. Ultrason Sonochem.

[23] Kheradmandnia S, Vasheghani-Farahani E, Nosrati M, Atyabi F (2010). The effect of process variables on the properties of ketoprofen loaded solid lipid nanoparticles of beeswax and carnauba wax. Iran J Chem Chem Eng.

[24] Jalilian M, Derakhshandeh K, Kurd M, Lashani H (2021). Targeting solid lipid nanoparticles with anisamide for docetaxel delivery to prostate cancer: preparation, optimization, and in-vitro evaluation. Iran J Pharm Res.

[25] Upadhyay SU, Patel JK, Patel VA, Saluja AK (2012). Effect of different lipids and surfactants on formulation of solid lipid nanoparticles incorporating tamoxifen citrate. J Pharm Bioallied Sci.

[26] Huynh L, Leroux JC, Allen C (2009). Enhancement of docetaxel solubility via conjugation of formulation-compatible moieties. Org Biomol Chem.

